# Chest sonography: a useful tool to differentiate acute cardiogenic pulmonary edema from acute respiratory distress syndrome

**DOI:** 10.1186/1476-7120-6-16

**Published:** 2008-04-29

**Authors:** Roberto Copetti, Gino Soldati, Paolo Copetti

**Affiliations:** 1 Emergency Department S. Antonio Abate General Hospital, Tolmezzo, Italy; 2 Emergency Department Valle del Serchio General Hospital, Lucca, Italy

## Abstract

**Background:**

Differential diagnosis between acute cardiogenic pulmonary edema (APE) and acute lung injury/acute respiratory distress syndrome (ALI/ARDS) may often be difficult. We evaluated the ability of chest sonography in the identification of characteristic pleuropulmonary signs useful in the diagnosis of ALI/ARDS and APE.

**Methods:**

Chest sonography was performed on admission to the intensive care unit in 58 consecutive patients affected by ALI/ARDS or by acute pulmonary edema (APE).

**Results:**

Ultrasound examination was focalised on finding in the two groups the presence of: 1) alveolar-interstitial syndrome (AIS) 2) pleural lines abnormalities 3) absence or reduction of "gliding" sign 4) "spared areas" 5) consolidations 6) pleural effusion 7) "lung pulse".

AIS was found in 100% of patients with ALI/ARDS and in 100% of patients with APE (p = ns). Pleural line abnormalities were observed in 100% of patients with ALI/ARDS and in 25% of patients with APE (p < 0.0001). Absence or reduction of the 'gliding sign' was observed in 100% of patients with ALI/ARDS and in 0% of patients with APE. 'Spared areas' were observed in 100% of patients with ALI/ARDS and in 0% of patients with APE (p < 0.0001). Consolidations were present in 83.3% of patients with ALI/ARDS in 0% of patients with APE (p < 0.0001). A pleural effusion was present in 66.6% of patients with ALI/ARDS and in 95% of patients with APE (p < 0.004). 'Lung pulse' was observed in 50% of patients with ALI/ARDS and in 0% of patients with APE (p < 0.0001).

All signs, except the presence of AIS, presented a statistically significant difference in presentation between the two syndromes resulting specific for the ultrasonographic characterization of ALI/ARDS.

**Conclusion:**

Pleuroparenchimal patterns in ALI/ARDS do find a characterization through ultrasonographic lung scan. In the critically ill the ultrasound demonstration of a dyshomogeneous AIS with spared areas, pleural line modifications and lung consolidations is strongly predictive, in an early phase, of non-cardiogenic pulmonary edema.

## Introduction

Acute respiratory distress syndrome (ARDS) and acute lung injury (ALI) are clinical syndromes characterized by inflammatory pulmonary edema, severe hypoxemia, stiff lungs, and diffuse endothelial and epithelial injury[1]. ALI/ARDS is associated most often with sepsis syndrome, aspiration, primary pneumonia, or multiple trauma and less commonly with cardiopulmonary bypass, multiple transfusions, fat embolism, pancreatitis, and other more rare causes.

In 1994 the American European Consensus Conference (AECC) established the three-criteria necessary for the definition of ALI and ARDS: 1) acute onset, bilateral infiltrates on chest radiography 2) pulmonary-artery wedge pressure less than 18 mmHg or the absence of clinical evidence of left atrial hypertension 3) PaO_2_/FiO_2 _ratio ≤ 300 for ALI and ≤ 200 in ARDS [2].

Before the emergence of computed tomography (CT) scanning for the study and characterization of this pathology, it was believed that ARDS affected equally all parts of the lung. The increased use of CT has led to a better understanding, diagnosis and management of ALI/ARDS. Study of the lung through CT scanning allowed a greater understanding of what happens to the acutely injured lung and showed it not to be uniformly involved: some areas of the lung are severely affected by an injury, some are mildly involved, and others are not involved at all. Alveolar filling, consolidation, and atelectasis occur predominantly in dependent lung zones, whereas other areas may be relatively spared [3,4]. Although greatly important in the initial characterization of patients with ALI/ARDS, CT scanning has many disadvantages. First of all it has the major disadvantage of exposing patients to high amounts of ionizing radiation. Secondarily, it is a costly resource and is not readily available in all hospital contests. Finally, it requires patient transport which, especially in hemodynamically unstable patients, always carries some risk [5].

Chest sonography has emerged in recent years as a very promising technique for the high sensibility it has shown in the detection of different lung and pleural pathological states [6-16].

Particularly, different studies have addressed the ultrasonographic appearance of ALI/ARDS but non have yet been able to give a detailed characterisization of the syndrome permitting a differential diagnosis from the ultrasonographic appearance of APE [12,17-19].

The purpose of this study was to evaluate the ability of chest sonography in the detection of characteristic pleuro-pulmonary signs of ALI/ARDS as compared to sonographic signs of APE.

### Chest sonography in alveolar-interstitial syndrome

Ultrasound lung comets (ULCs) are an ultrasonographic sign of subpleural interlobular septal thickening either due to hydrostatic edema, as in pulmonary edema, or to connective tissue, as in pulmonary fibrosis [10]. Their absolute number is strictly correlated with the entity of extravascular lung water [20-24].

Normal lung appears on chest ultrasonography as "black", moderately diseased lung with the presence of increased interstitial water as "black and white" and markedly diseased lung, with the presence of greatly increased extravascular lung water and alveolar edema, as "white" (diffusely bright) [23].

ULCs therefore identify alveolar-interstitial syndrome.

Echographic characterization of alveolar-interstitial syndrome is simple and its recognition does not require sophisticated technology. Bedetti et al. demonstrated a high correlation betweeen AIS assessment obtained by experienced echocardiologists using a full feature echocardiographic platform and by inexperienced sonographers, after a very limited (30') time dedicated to training, using a hand-held echocardiography system [24].

In our hypothesis the different pathophysiology working respectively in ALI/ARDS and in cardiogenic (hydrostatic) pulmonary edema (APE) produces different pleuropulmonary sonographic patterns with a different distribution of AIS.

## Materials and methods

From January 2005 to April 2007, 18 among the patients consecutively admitted to the intensive care unit of our hospital fulfilled the American-European Consensus Conference diagnostic criteria for the diagnosis of ALI/ARDS[2]. During the same period 40 patients were consecutively admitted with the diagnosis of acute pulmonary edema (APE) on the base of clinical signs and symptoms, electrocardiogram, chest x-ray, and Color-Doppler echocardiography.

Informed consent and approval of ethic committee were not requested since lung ultrasonography is part of routine diagnostic procedures in our unit.

Tables 1 and 2 show the demographic characteristics of patients with ALI/ARDS and APE.

**Table 1:** Demographic characteristics and major clinical information of patients with ALI/ARDS

**ALI/ARDS (n = 18)**	
**AGE (y)**	
Mean ± SD	68 ± 11.2
Range	47–80
**Sex (no. of patients)**	
M	11
F	7
**Cause of ALI/ARDS (no. of patients)**	
Acute pancreatitis	3
Sepsis	10
Fat embolism	1
Aspiration	2
Pneumonia	2
ALI	3
ARDS	15
Mechanical ventilation	16
Non invasive ventilation	2

**Table 2:** Demographic characteristics and major clinical information of patients with APE

**APE (n = 40)**	
**AGE (y)**	
Mean ± SD	75.5 ± 6.8
Range	61–84
**Sex (no. of patients)**	
M	25
F	15
**Cause of APE (no. of patients)**	
Systolic dysfunction	20
Diastolic dysfunction	12
Valvular disease	8
Mechanical ventilation	8
No invasive ventilation	32

A convex probe 3.5–5 MHz and linear probe 7.5–10 MHz (Megas CVX Esaote Medical Systems, Florence-Italy) were used for lung examination. The exam was performed at patient bedside. Longitudinal and transversal scans of the anterior, lateral and posterior wall were performed. Lateral or seated positions were used to scan the posterior thorax. In patients in whom the seated position was not possible a lateral decubitus position was used to examine posterior lung regions.

Each hemithorax was divided into 5 areas: 2 anterior, 2 lateral and 1 posterior. The anterior chest wall was delineated from the parasternal to the anterior axillary line and was divided into upper and lower halves (from the clavicle to the second-third intercostal space and from the third space to the diaphragm). The lateral area was delineated from the anterior to the posterior axillary line and was divided into upper and basal halves. The posterior area was considered as the zone beyond the posterior axillary line [7,16].

A sector probe 2–3.5 MHz (Megas CVX Esaote Medical Systems, Florence-Italy) was used for an echocardiographic ultrasound examination aimed at the study of left ventricular systolic and dyastolic function.

Chest sonography was performed in all patients on the first day of admission and after having obtained a clinical and radiologic diagnosis of disease. Ultrasound pleuropulmonary findings were considered with respect to the presence of the following signs:

1) Alveolar-interstitial syndrome (AIS) defined as the presence of more than 3 ULCs or "white lung" appearance for each examined area.

2) Pleural lines abnormalities defined as thickenings greater than 2 mm, evidence of small subpleural consolidations or coarse appearance of the pleural line.

3) Areas with absent or reduced "sliding" sign with respect to adjacent or controlateral zones at the same level on the opposite hemithorax.

4) "Spared areas" defined as areas of normal lung pattern in at least one intercostal space surrounded by areas of AIS.

5) Consolidations defined as areas of hepatisation (tissue pattern) with presence of air bronchograms [25].

6) Pleural effusion defined as anechoic dependent collections limited by the diaphragm and the pleura [25].

7) "Lung pulse" [6] defined as absence of lung sliding with the perception of heart activity at the pleural line.

### Statistical analysis

All data were analyzed with SPSS for Windows, version 14.0 (Chicago, IL). The demographic variables were compared by two tailed Student t tests. Continuously distributed variables were expressed as means ± SD. Categorical variables were presented as counts and percentages. Each of the seven sonographic findings in the two types of edema was compared by a chi-squared test. We accepted a p value < 0.01 as statistically significant.

## Results

The total study population included 58 patients. 18 met the criteria for the diagnosis of ALI/ARDS and 40 had APE. Males were 36 (62%). The two groups were similar for age and sex.

**Figure 1:**
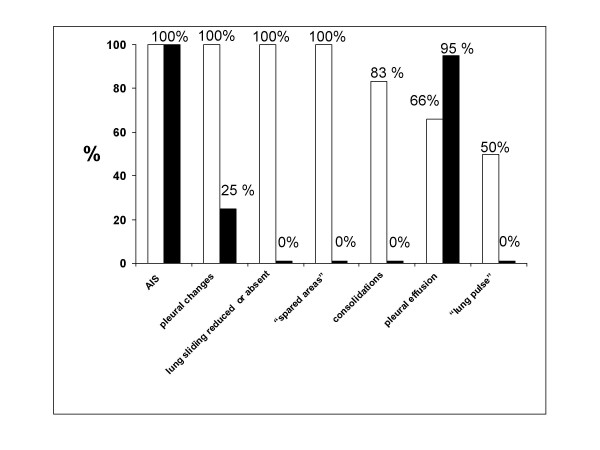
Percentage of the different signs in the two groups.

Table 3 shows the distribution of ultrasound signs in the two studied groups, figure 1 the percentage of the different signs in the two groups and table 4 the sensitivity and specificity of each ultrasonographic sign in the two groups.

**Table 3:** Distribution of ultrasound signs in the two studied groups

	ALI/ARDS (18 pt.)	APE (40 pt.)	p
Sex (n° males)	11 (61%)	25 (62.5%)	0.92 (ns)
Mechanical ventilation (n)	16 (88.8%)	8 (20%)	< 0.0001
AIS	18 (100%)	40 (100%)	ns
Pleural line abnormalities	18 (100%)	10 (25%)	< 0.0001
Reduction or absence of lung sliding	18 (100%)	0	< 0.0001
"Spared areas"	18 (100%)	0	< 0.0001
Consolidations	15 (83.3%)	0	< 0.0001
Pleural effusion	12 (66.6%)	38 (95%)	0.004
"Lung pulse"	9 (50%)	0	< 0.0001

**Table 4:** Sensitivity and specificity of each ultrasonographic sign in the two groups.

SONOGRAPHIC SIGNS	SENSITIVITY		SPECIFICITY	
	
	ALI/ARDS	APE	ALI/ARDS	APE
AIS	100%	100%	0%	0%
Pleural line abnormalities	100%	25%	45%	0%
Reduction or absence of lung sliding	100%	0%	100%	0%
"Spared areas"	100%	0%	100%	0%
Consolidations	83.3%	0%	100%	0%
Pleural effusion	66.6%	95%	5%	33.3%
"Lung pulse"	50%	0%	100%	50%

### Analysis of ultrasound picture of ALI/ARDS and APE

**Figure 2:**
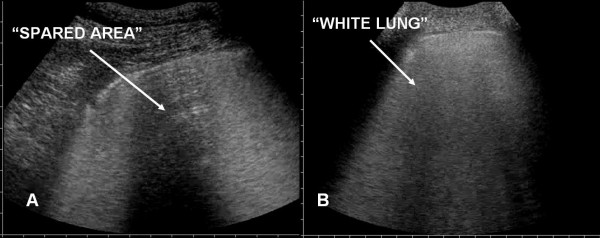
Spared areas: present in ARDS (panel A), absent in APE (panel B).

In all of the patients with ALI/ARDS the lung ultrasonographic picture resulted characteristic. In the anterior lung fields there was constant evidence of bilateral, not homogeneously distributed AIS. In some areas ULCs were numerous, in others they were compact, and between these two there were areas of normal lung ("spared areas") configuring a spotted distribution of AIS (Fig. 2).

In the posterior lung fields AIS appeared more homogeneous showing the presence of compact ULCs that produce an echographic "white lung".

**Figure 3:**
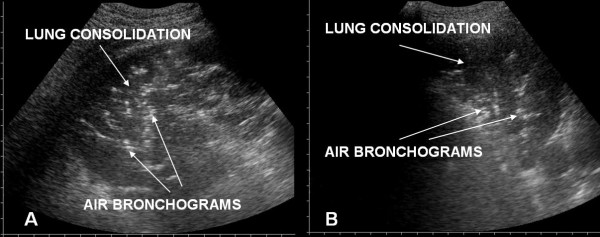
Lung consolidations with air bronchograms in posterior lung fields in ARDS (panel A and B).

Areas of consolidation were often present in posterior fields, especially at the bases, with evidence of static or dynamic air bronchograms.(Fig. 3).

**Figure 4:**
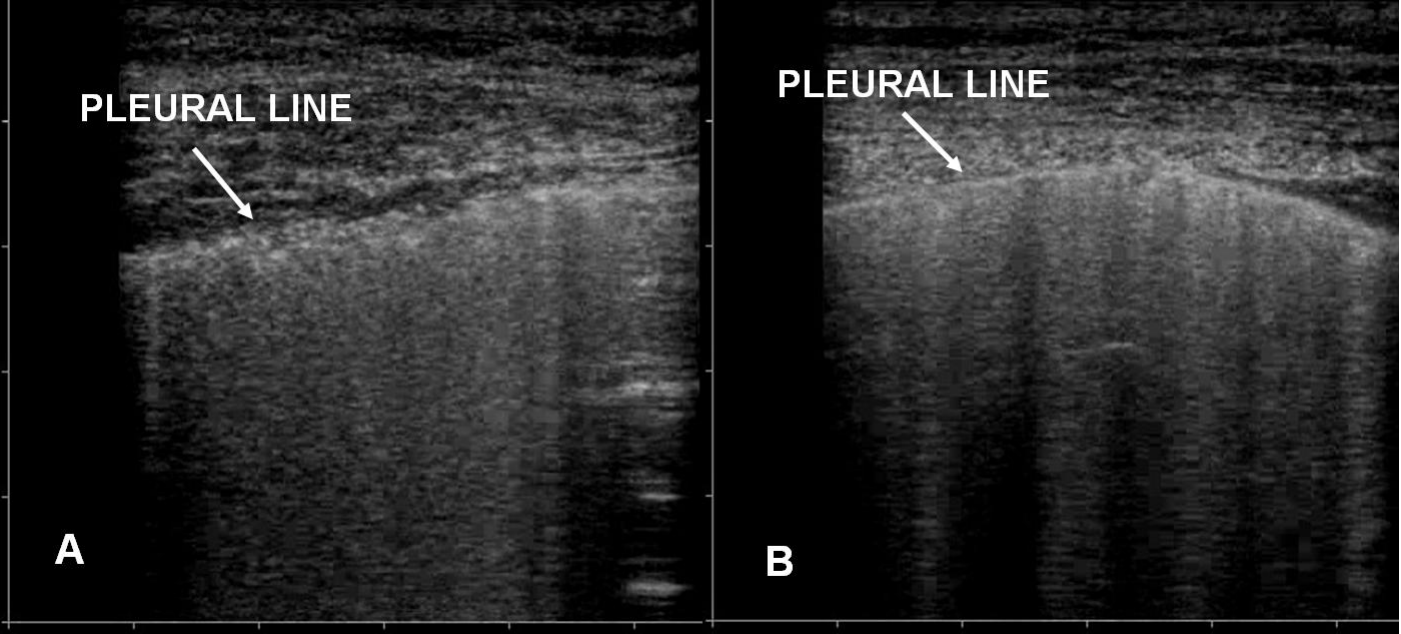
Pleural line: altered in ARDS (panel A), normal in APE (panel B).

**Figure 5:**
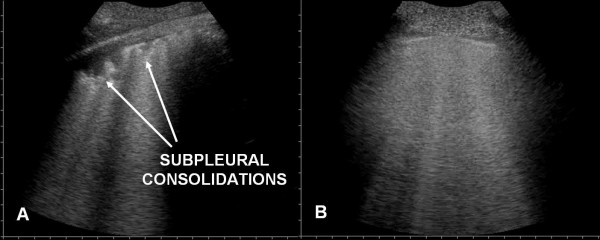
Small subpleural consolidations: present in ARDS (panel A), absent in APE (panel B).

**Figure 6:**
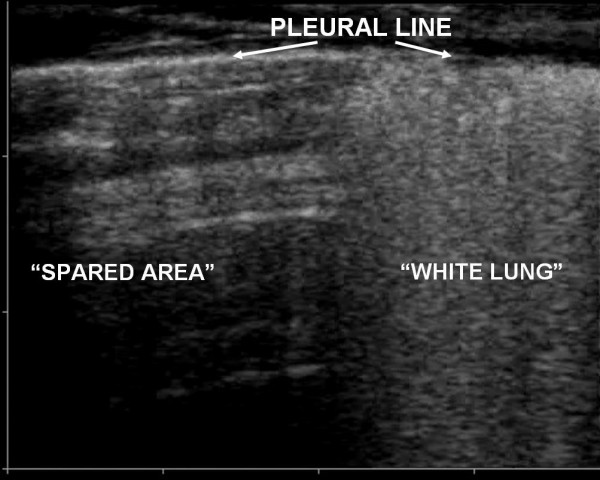
Particular of pleural line with linear probe (10 MHz): above the spared area the pleural line is normal while it is altered above the area of AIS.

The pleural line was constantly involved. "Lung sliding" was reduced or absent and often in the areas in which ULCs were compact the "lung pulse" sign could be observed. The pleural line appeared irregular, thickened and coarse for the presence of multiple small subpleural consolidations. Involvement of the pleural line was not homogeneous and followed faithfully the distribution and the degree of the AIS (Fig. 4, 5 and 6).

**Figure 7:**
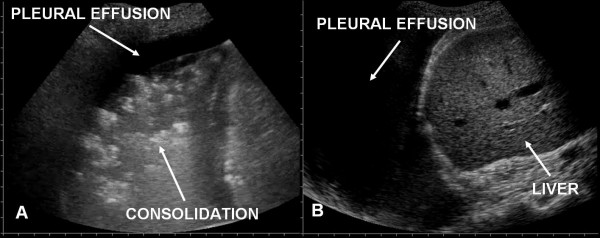
Pleural effusion: small pleural effusion in ARDS (panel A), larger pleural effusion in APE (panel B).

Pleural effusions were not frequent (Fig. 7).

Ultrasonographic lung appearance did not differ between patients with ALI and those with ARDS nor between primary and secondary ALI/ARDS.

Sonographic appearance of APE is characterised by AIS homogeneously involving both anterior and posterior lung fields. Superior lung fields might be less affected, but "spared areas" were never observed (Fig. 2). The pleural line was rarely involved, and appeared as a hyperechoic band without sliding impairment (Fig. 4, 5). Small subpleural consolidations were found in some cases, particularly at the posterior bases. Pleural effusions were common and usually larger than in ALI/ARDS. (Fig. 7).

AIS was present in the entire study population confirming its already established high sensitivity in the diagnosis of increased extravascular lung water but resulting to be an aspecific sign present in both ALI/ARDS and APE.

All other signs considered demonstrated a statistically significant difference in their occurrence in the two different study groups (Table 3).

## Discussion

The definition of ALI/ARDS of the AECC is simple to apply in the clinical setting but there are some problems concerning two of three criteria proposed: (a) radiographically the findings are hardly distinguishable from those of cardiogenic pulmonary edema in that chest radiography, even if it sometimes shows certain features, is generally inaccurate [26,27] (b) clinical assessment of left atrial hypertension is not easy and measurement of pulmonary-artery wedge pressure requires pulmonary artery catheterization. Moreover a pulmonary artery occlusion pressure greater than 18 mmHg does not rule out the diagnosis of ALI. Patients with sepsis, for example, may develop ALI and severe left ventricular dysfunction (sepsis-related cardiomyopathy). Even when the pulmonary artery occlusion pressure is less than 18 mmHg, one cannot be certain that edema is the result of altered permeability. Furthermore, reduction of colloid oncotic pressure, as in hypoalbuminemic states, promotes edema in the absence of permeability changes[28].

Clinical history and the identification of the inciting clinical disorders associated with ALI and ARDS are often crucial in diagnostic orientation.

In cardiogenic pulmonary edema interstitial fluid flows with centripetal fashion progressively distending lymphatic vessels and engorging peribronchial tissue. This happens in the thick interstitium when alveolar membrane thickness is normal, thus before alveolar flooding takes place. Only when the drainage ability of the lymphatic system is overcome alveolar flooding takes place with the "all or none" phenomenon: each single alveolus is either filled with fluid or filled with air [29,30].

In ALI/ARDS the integrity of the alveolar capillary membrane is compromised, and this causes an early, diffuse, heterogeneous alveolar flooding which ranges in severity from "ground glass" appearance to lung consolidation.

Air bronchograms and relatively spared areas are commonly detected [31,32,1]. Moreover in ARDS the edema safety factor decreases by about half, and flooding develops at lower capillary hydrostatic pressure [33].

Physiopathologic differences between APE and ALI/ARDS account for the differences observed echographycally.

CT allows the distinction of interstitial edema from that involving air spaces better than traditional radiology [34]. In the former, interlobular septa are thickened, as are subpleural areas and the peribronchovascular connective tissue; in the latter CT shows "ground glass" areas produced by alveolar-interstitial edema or alveolar consolidations with air bronchograms in case of massive alveolar flooding. Unfortunately chest CT can not be performed at bedside and furthermore it has a high radiation exposure, equivalent to 400–500 chest X-rays.

Our results demonstrate that chest sonography represents a useful tool for the diagnosis because it can detect very peculiar findings.

Ultrasound AIS is a marker of pulmonary edema being present both in ALI/ARDS and APE patients [35].

Our results confirm that ultrasound evaluation of the increased extravascular lung water is feasible at the bedside [20-24].

According to our data AIS as described by Lichtenstein[10] in 1997 can not be considered a singular ultrasound entity. It can be distinguished not only trough ULCs density (black-white or white) but even on the basis of the homogeneity of the AIS and the presence of other related signs [36,37]. We actually observed lung "spared areas" only in ALI/ARDS. The heterogeneous involvement of the lungs in ALI/ARDS explains the presence of spared areas. Furthermore, presence of posterior lung consolidations with air bronchograms is typical of ALI/ARDS.

It is worth noting that pleural line abnormalities were always present in ALI/ARDS, particularly reduction/absence of pleural gliding, thickening and coarse appearance of pleural line and lung pulse. On the contrary, in acute cardiogenic pulmonary edema alveolar-interstitial syndrome shows a homogeneous distribution, pleural line is regular, "lung sliding" is normal and lung consolidations are not characteristic. Although pleural effusions resulted being more frequently present in APE than in ALI/ARDS, their presence cannot be relied on for differential diagnosis.

Pleural line alterations, reduction or absence of pleural gliding and areas where the "lung pulse" is present, are all better evaluated with a linear, high frequency probe being the pleural line superficial. Use of sector of microconvex probes could make the observation of these alterations difficult since only a smaller part of pleural line is able to be observed. In our study a linear probe was constantly used for the study of the pleural line.

In our study a large number of patients with ALI/ARDS underwent mechanical ventilation.

In many cases chest sonography was performed before intubation and no change was immediately observed after initiation of mechanical ventilation. Changes in consolidation areas, usually more evident in the posterior lung fields, are observed after recruitment maneuvers. This may suggest a role of ultrasound not only in the diagnosis of ALI/ARDS but also for a bedside evaluation of lung recruitment during ventilatory challenges through evidence of ventilation and re-expansion in consolidated or atelectasic areas.

We often observed, in patients with ALI/ARDS, areas of pleural line showing the "lung pulse" sign previously described in the presence of complete atelectasis. This sign is very peculiar and could be the result of lung consolidations possibly masked by AIS or be caused by a critical reduction in pulmonary compliance.

Often the differentiation of ALI/ARDS from cardiogenic pulmonary edema may be very difficult in postoperative patients with fever and high markers of inflammation. In these situations chest radiography is inaccurate, CT is expensive, biologically invasive and not portable, and lung ultrasound may result crucial for a correct interpretation of chest radiography.

Beyond interpreting CT scans, a more repeatable use of chest sonography offers new perspectives for an early diagnosis of permeability edema, its differential from cardiogenic edema and generic chest monitoring of the critically ill.

In fact, in a case of interstitioalveolar syndrome, we think that showing pleural line irregularity, lung pulse, and especially alveolar consolidations with spared areas, is strongly predictive of permeability edema.

Future validations of our data could limit CT use in ICU and a precocious diagnosis of permeability pulmonary edema could be performed by detecting specific ultrasonographic findings.

## Conclusion

Chest sonography has recently found an extensive use in pleuropulmonary disorders in Emergency and ICU settings. Experimental and clinical evidence supports its usefulness in detecting pulmonary edema. Ultrasonographic appearance of interstitial and alveolar-interstitial syndromes differs according to the underlying disorder. Pleuroparenchimal patterns in ALI/ARDS accurately described by CT scan do find a characterization even through ultrasonographic lung scan. In critically ill patients ultrasound demonstration of a dyshomogeneous AIS with spared areas, pleural line modifications and lung consolidations is strongly predictive, in an early phase, of a non cardiogenic pulmonary edema.

## Authors' contributions

RC and GS conceived this study. RC and PC performed chest sonography in patients with ALI/ARDS and APE. GS performed the statistical analysis of the study. All authors read and approved the final manuscript.
